# 

**Published:** 1891-05

**Authors:** 


### TABLE OF CONTENTS.

ORIGINAL COMMUNICATIONS. PAGE

The Formation of Enamel. By R. R. ANDREWS, D.D.S. . . . . . . . . 273

Plastic Materials in the Preparatory Treatment of Teeth. By JACOB L. WILLIAMS, M.D. . . . . . . . . . . . . . . . . . . . . . . . . . 281

Work. By PROFESSOR J. S. WIGHT, M.D. . . . . . . . . . . . . . . 284

Office Notes. By DR. WILLIAM H. ROLLINS . . . . . . . . . . . . . 292

Removal of the Pulp by the Use of Cocaine. By EDWARD C. BRIGGS, M.D., D.M.D. . . . . . . . . . . . . . . . . . . . . . . . . . . 296

REPORTS OF SOCIETY MEETINGS.

New York Odontological Society . . . . . . . . . . . . . . . . . . 300

Odontological Society of Pennsylvania . . . . . . . . . . . . . . . 307

American Academy of Dental Science . . . . . . . . . . . . . . . . 319

Harvard Odontological Society . . . . . . . . . . . . . . . . . . . 323

EDITORIAL.

Contour Fillings: Their Place in Dental History . . . . . . . . . . . 332

William H. Atkinson, M.D., D.D.S. . . . . . . . . . . . . . . . . . 334

Bibliography . . . . . . . . . . . . . . . . . . . . . . . . . . . 335

OBITUARY.

Daniel Frank Whitten, D.M.D. . . . . . . . . . . . . . . . . . . . 336

William H. Atkinson, M.D., D.D.S. . . . . . . . . . . . . . . . . . 336

Dr. Ambler Tees, Sr. . . . . . . . . . . . . . . . . . . . . . . . 336

CURRENT NEWS.

The Dental Protective Association of the United States . . . . . . . . 338

Items . . . . . . . . . . . . . . . . . . . . . . . . . . . . . . 340

